# *Cohnella* amylopullulanases: Biochemical characterization of two recombinant thermophilic enzymes

**DOI:** 10.1371/journal.pone.0175013

**Published:** 2017-04-10

**Authors:** Fatemeh Zebardast Roodi, Saeed Aminzadeh, Naser Farrokhi, AliAsghar Karkhane, Kamahldin Haghbeen

**Affiliations:** 1 Department of Industrial and Environmental Biotechnology, National Institute for Genetic Engineering and Biotechnology (NIGEB), Tehran, Iran; 2 Department of Biotechnology Engineering, Faculty of New Technologies Engineering, Shahid Beheshti University G.C., Tehran, Iran; Kermanshah University of Medical Sciences, ISLAMIC REPUBLIC OF IRAN

## Abstract

Some industries require newer, more efficient recombinant enzymes to accelerate their ongoing biochemical reactions in harsh environments with less replenishment. Thus, the search for native enzymes from extremophiles that are suitable for use under industrial conditions is a permanent challenge for R & D departments. Here and toward such discoveries, two sequences homologous to amylopullulanases (EC 3.2.1.41, GH57) from an endogenous *Cohnella* sp., [*Coh00831* (KP335161; 1998 bp) and *Coh01133* (KP335160: 3678 bp)] were identified. The genes were heterologously expressed in *E*. *coli* to both determine their type and further characterize their properties. The isolated DNA was PCR amplified with gene specific primers and cloned in pET28a, and the recombinant proteins were expressed in *E*. *coli* BL21 (DE3). The temperatures and pH optima of purified recombinants *Coh 01133* and *Coh 00831* enzymes were 70°C and 8, and 60°C and 6, respectively. These enzymes are stable more than 90% in 60°C and 50°C for 90 min respectively. The major reactions released sugars which could be fractionated by HPLC analysis, from soluble starch were mainly maltose (G2), maltotriose (G3) and maltotetraose (G4). The enzymes hydrolyzed pullulan to maltotriose (G3) only. Enzyme activities for both proteins were improved in the availability of Mn^2+^, Ba^2+^, Ca^2+^, and Mg^2+^ and reduced in the presence of Fe^2+^, Li^2+^, Na^2+^, Triton X100 and urea. Moreover, Co^2+^, K^+^, and Cu^2+^ had a negative effect only on *Coh 01133* enzyme.

## Introduction

Costs associated with food and beverage enzymes are expected to reach $1.7 billion US dollars by 2018 [1]. Among the diverse relevant enzymes, starch hydrolyzing enzymes (namely α-amylases, α-amylase-pullulanases, and amylopullulanases) have found their way into starch and baking industries as catalysts [2]. These enzymes fall into two families of glycosyl hydrolases, GH13 and GH57, and both having four conserved regions that classify them within the family of α-amylases [3, 4]. Pullulanases are being used in industrial applications, such as one-step liquefaction-saccharification towards the production of sugar syrups and as an anti-staling agent in the baking industry, and are mainly produced by thermophilic bacteria and archaea [2, 3]. This enzyme not only has application in food and beverage industries, it is being used within ethanol, detergents, dishwashing, laundry detergents, textile, and pulp and paper industries as well. Starch enzymatic alteration into sugar syrups is done at high temperature, in liquefaction, and saccharification steps follows at 60°C. Furthermore starch bioprocessing at higher temperature improves starch solubility, restricts microbial contamination, reduces its viscosity, decreases reaction times and more economical [5]. Therefore it seems necessity to discover suitable thermoactive and thermostable enzyme which improves saccharification rate and the other process yield [6]. Amylopullulanases (EC 3.2.1.41) are classified into two groups: Type I that cleaves α-(1→6) linkages in pullulan and branched oligosaccharides such as amylopectin, producing maltotriose and unbranched oligosaccharides, and Type II pullulanase or amylopullulanase that has both amylase and pullulanase activity [7, 8]. The latter cleaves starch glycosidic bonds at both α-(1→6) and α-(1→4) linkages, releasing a remaining polysaccharide. In contrast, α-amylase cleaves only α-(1→4) linkages in varieties of substrates such as starch, glycogen, and cyclodextrins [9]. According to the number of active sites within the enzyme amylopullulanase can be divided into two subgroups [10]. Most thermophilic amylopullulanases possess one active site [11].

Here, two amylopullulanases from an endogenous *Cohnella* sp. A01 toward their identification and biochemical characterization were heterologously expressed in *E*. *coli* in the search for industrial enzymes. The enzyme features were compared with some previously known orthologs, and phylogenetic analysis were carried out to define their relatedness to either GH13 or GH57.

## Materials and methods

### Materials

*Taq* DNA polymerase, *Bam*H I, *Nhe* I, *Nde* I, *Sal* I, T4 DNA ligase, and IPTG were purchased from Thermo Scientific™. Strains including *E*. *coli* DH5α and BL21 (DE3), pET-28a vector and Ni-NTA resin were purchased from Invitrogen (Carlsbad, USA). Ampicillin (A0166-5G), kanamycin (K1377-5G), agarose (A9539-50G), pullulan (91335), and starch (S2630) were acquired from Sigma (Steinheim, USA). The DNA extraction kit was obtained from BioNEER (Seoul, Korea). HPLC-grade water and acetonitrile gradient grade for liquid chromatography were purchased from Merck. Other materials were purchased from Merck (Darmstadt, Germany) and Sigma (St. Louis, USA). *Cohnella* sp. A01 was obtained from sampled water of a shrimp pond at Choebdeh (Abadan, Iran) and further characterized by microbiological and 16s rRNA sequencing (Accession No. JN208862.1) [12]. All experiments were performed with at least three replicates, unless otherwise stated.

### Bioinformatic analysis

Amylopullulanase and α-amylase protein sequences of GH13 and GH57 were obtained from CaZy (http://www.cazy.org; Table A in S1 File). Multiple sequence alignment was performed with the sequences using ClustalW. A phylogenetic tree was established using neighbor-joining with 1000× bootstrap value in MEGA5 [13]. Relative molecular weights and isoelectric points were predicted using ProParam at ExPASy (http://web.expasy.org/protparam/) [10].

### Cloning and expression of amylopullulanases

A single clone of *Cohnella* sp. A01 was cultured in NB at 60°C for 72 h. Genomic DNA was isolated using a DNA extraction kit. *Coh 01133* gene (3678 bp) and *Coh 00831* gene (1998 bp) were PCR amplified using two gene specific primer pairs containing properly engineered restriction sites that were used for following restrictions and ligations as below:

*Coh00831*:

Fcoh00831: 5'CTTGCTAGCATGAAACGGGTTCCAAACCTGC3'; *Nhe* I; T_m_: 59°C and Rcoh00831: 5'CTTGGATCCTAATTGGTGAAGCCTGCCGAGC3'; *Bam H* I; T_m_: 60°C.

*Coh01133*:

Fcoh01133: 5'CTTCATATGCTCCGTCGGGAGCGGAAGTGCGGATCGC3'; *Nde* I; T_m_: 77°C and Rcoh01133: 5'GGA*GTCGAC*TCAGGATTTCAATAATTCCGCGTACTC3'; *Sal* I; T_m_: 54°C.

The amplicons were digested with *Bam*H I and *Nhe* I in *Coh 00831*, and *Nde* I and *Sal* I in *Coh 01133*. The restricted fragments were cloned in pET28a using 1 U T4 DNA ligase and transformed in BL21 (DE3) [14]. The T_7_ promoter was induced with 1 mM IPTG for 4 h at 22°C.

### Purification of recombinant amylopullulanases

*Coh 01133* amylopullulanase was purified with Ni-NTA Sepharose affinity chromatography at 4°C according to the Invitrogen protocol. *Coh 00831* amylopullulanase was purified via anion exchange chromatography using DEAE-Sepharose column (2 × 20 cm). A single step ion exchange chromatography method has been developed for purification of *Coh 00831* amylopullulanase. The crude extract was dissolved in phosphate buffer (10 mM) having a pH between 6 and 8 and with an increase of pH 0.5. The samples were added to an anion exchange matrix using DEAE-Sepharose equilibrated with 10 mM phosphate buffer at the corresponding test pH. The amount protein and amylopullulanase activity of the supernatant were determined. The buffer ionic strength effect on adsorption was also specified by varying concentrations of phosphate (10, 20, 30 and 40 mM) at pH 8. NaCl solution optimum ionic strength was determined by eluting the adsorbed protein using a 0.1 to 1.5 M solution.

After finding the optimum parameters, DEAE-Sepharose column equilibrated with 20 mM phosphate buffer, pH = 8.0 [15, 16]. The proteins were eluted with 0–0.5 M NaCl gradient, and eluents were monitored at 280 nm and further tracked via SDS-PAGE. Both proteins were dialyzed against a 50 mM sodium acetate buffer (pH = 5.5).

### Recombinant amylopullulanases enzyme assay

Amylopullulanase activity assay was performed using spectrophotometric method based on Millers method [17] while pullulan and starch were used as enzyme substrate. Production of reducing sugar in the reaction mixture shows the enzyme activity by addition of 3,5-dinitrosalicylic acid (DNS). The reaction mixture (0.5 ml) containing 1% (w/v) substrate solution, 50 mM sodium acetate buffer (pH = 5.5), and an enzyme source was incubated at 60°C for 30 min. One unit of pullulanase activity is defined as the amount of enzyme which produces 1 μmol of reducing sugar (with glucose as the standard) per minute under assay conditions.

pH profiles were performed in 50 mM mixed buffer containing sodium phosphate, sodium acetate, and glycine. For pH stability, enzymes were incubated for 90 min at pH = 3–12 (5mM mixed buffer) and the relevant activities were monitored at 540 nm; data was presented using Prism5.

Furthermore, in the temperature profile, relative enzyme activities were determined in temperatures ranging 20–100°C. Meanwhile, enzymes were incubated at 20–100°C for 90 min at their optimum pHs. The heat-treated enzymes were used for assay. Following the addition of DNS, enzyme activities were determined at 540 nm to perform temperature stabilities. The relevant graphs were prepared with Prism5.

The products from soluble starch and pullulan hydrolyzed by the amylopullulanases were examined with HPLC (Agilent 1260 A) using an Aminex HPX-87H column (Bio-Rad, USA) connected to a RID detector. The products were eluted with a mixture of acetonitrile: double-deionized HPLC-grade water (60: 40 [V/V]) as solvent at a flow rate of 1 ml per min and column temperature of 20°C.

The effects of 5 mM metal cations (MgCl_2_, CaCl_2_, KCl, NiCl_2_, LiCl_2_, FeCl_2_, MnCl_2_, ZnCl_2_, BaCl_2_, CuCl_2_, CoCl_2_, and NaCl), detergents (0.1% tween 20, 6 M urea, 1 mM SDS, and 0.1% Triton X100), 2 and 5 mM EDTA as chelator, and 10 mM iodoacetic acid as reducing agent on the recombinant enzymes activities were analyzed. Enzymes (50 μl) at their pH optima were added to mixtures of 50 μl 1% pullulan and each of above-mentioned chemicals. The reactions were incubated at optimum temperatures for 30 min.

Kinetic parameters for enzyme catalysis for both enzymes using pullulan and starch as substrates were obtained by Prism5.

### Zymogram analysis

Isolated recombinant enzymes were separated on a 10% native-PAGE containing 1% (w/v) pullulan. The gel was washed with 2.5% Triton X100 and incubated for 1 h with the proper buffer providing optimum pH for each enzyme, 50 mM either sodium acetate (pH = 6.0) or sodium phosphate (pH = 8.0), at 4°C. The gels were incubated at 60°C for 1 h; then Lugol’s reagent was added to the gel, and enzyme activity was photographed.

## Results

### Bioinformatic analysis

**Fig 1:**
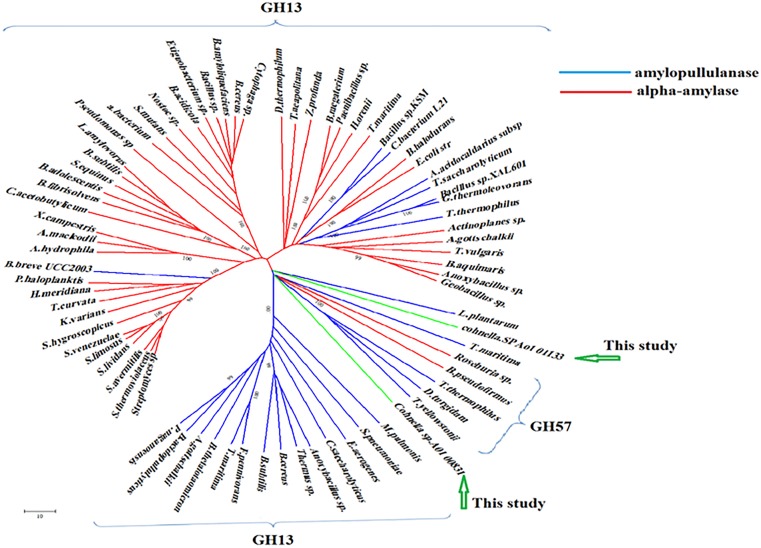
Phylogenetic analysis of bacterial α-amylases and amylopullulanases, including both *Coh01133* and *Coh00831*. The tree illustrates that the two *Cohnella* amylopullulanases are well-grouped within GH57, residing close to other functionally characterized amylopullulanases. Phylogenetic tree was established using neighbor-joining with 1000× bootstrap value in MEGA5. The list of protein sequences and their accession numbers are provided in Table A in S1 File.

Bacterial GH13 and GH57 (http://www.cazy.org) proteins, representative members of the amylopullulanases and α-amylases families, were collected and run through MEGA5. The four conserved motifs that have been reported to be the main scaffolds for α-amylase family of enzymes at positions of: I: 316-AADGSV-S, II: 383-EVRLDKV, III: 408-YEAWR and IV: 507-VDGTDK in *Coh 00831* (KP335161 GenBank accession no.) amino acid sequence and I: 567–GTSPLVDLSGNA, II: 670- SPALTDLA, III: 729- VEVNG and IV: 816- TDGEG in *Coh* 01133 (KP335160 GenBank accession no.) amino acid sequence were evident (Table 1) [3, 18, 19]. The deduced amino acid sequences of the amylopullulanases were compared with the sequences of the related enzymes available through GenBank (Table A in S1 File). *Cohnella* sp. A01 00831 amino acid sequence had 58% identity with the reported amylopullulanase from *D*. *turgidum*, while the highest percentage identity with *Cohnella* sp. A01 01133 amino acid sequence was noted with amylopullulanase from *M*. *pulmonis* (47%) (Table B in S1 File). Phylogenetic analysis of *Coh 01133* amino acid sequence and *Coh 00831* amino acid sequence within the bacterial tree indicated that these two proteins belong to a single clade grouped with GH57 (Fig 1). No significant similarity found between *Coh 00831* and *Coh 01133* amino acid sequences. *Coh 01133* and *Coh 00831* enzymes were predicted to have relative molecular weights of ~127 and ~70 kDa with isolectric points of 6 and 5.37, respectively.

**Table 1:** Conserved amino acid sequences in four motifs (I, II, III, IV) of pullulanases from this study and earlier reports.

	I	II	III	IV
*Cohnella* sp.A01 01133	567–GTSPLVDLSGNA	670- SPALTDLA	729- VEVNG	816- TDGEG
*Cohnella* sp.A01 00831	316-AADGSV-S	383-EVRLDKV	408-YEAWR	507-VDGTDK
*C*.*bacterium*L21-TH-D2	MVDVVINH	YFRVDTV	GETYG	LGSHDE
*Bacillus* sp.XAL601	ILDGVFNH	GWRLDVA	GEIWD	LGSHDT
*B*. *breve* UCC2003	VMDVVYNH	GFRFDLM	GEGWD	VEIHDN
*B*. *cereus*	IIDVVYNH	GFRFDLM	GEGWD	VECHDN
*T*. *saccharolyticum*	ILDGVFNH	GWRLDVE	AENWG	LGSHDT
*T*. *thermohydrosulfuricus*	ILDGVFNH	GWRLDVA	AENWN	LGSHDT
*A*. *gottschalkii*	IKDVVYNH	GFRFDLM	GEPWQ	VSCHDN
*Anoxybacillus* sp.LM18-11	ILDVVYNH	GFRFDLM	GEGWD	VECHDN
*T*. *maritima* MSBB	IMDMVFPH	GFRFDQM	GEPWG	AACHDN
*L*. *plantarum*	VMDMVLNH	TLNDSLW	PLSPTQ	SGNSVK
*M*. *pulmonis*	MDVVYNH	GFRFDLs	HGEAW	SACHDG
*D*. *turgidum* (GH57)	WIDGTFLT	DLAFDEL	GEEKP	ELLIDF

### Expression and purification of the recombinant pullulanase

**Fig 2:**
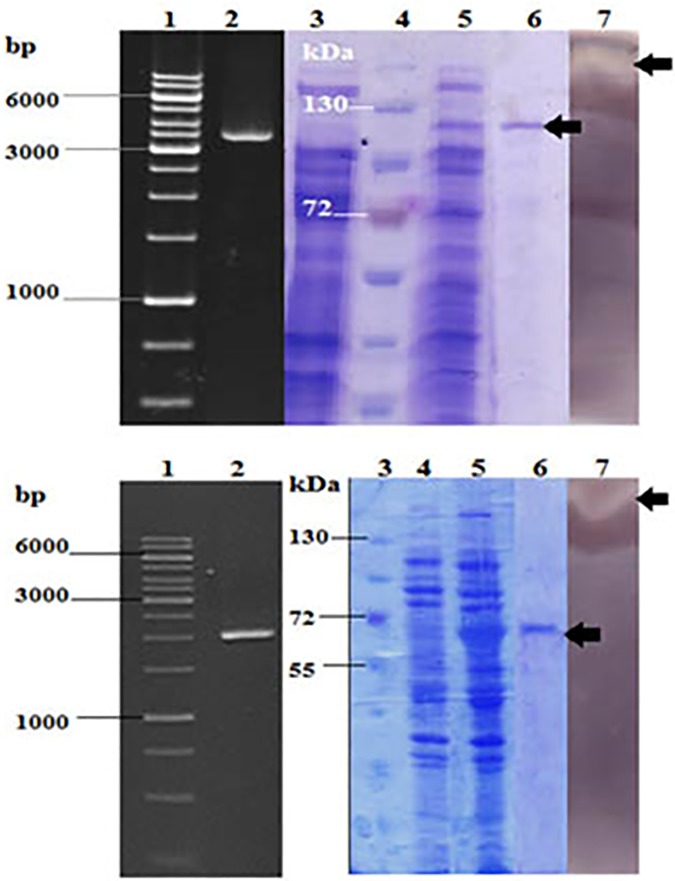
Polymerase chain reaction, heterologous gene expression and zymogram analysis of *Coh01133*. a) Lane1 = DNA marker ladder, Lane 2 = PCR amplicon of *Coh01133* separated on 1% agarose gel, Lane 3 = uninduced *Coh01133* construct, Lane 4 = protein ladder, Lane 5 = induced *Coh01133* construct, Lanes6 = Ni-NTA sepharose column washed with 250 mMImidazol, Lane 7 = Zymogram analysis of *Coh01133* expressed and purified proteins in a gel containing starch as substarte b) Lane1 = DNA marker ladder, Lane 2 = PCR amplicon of *Coh00831* separated on 1% agarose gel, Lane 3 = protein ladder, Lane 4 = uninduced *Coh00831* construct, Lane 5 = induced *Coh00831* construct, Lanes 6 = purified *Coh00831* with ion exchange chromatography, Lane 7 = Zymogram analysis of *Coh00831* expressed and purified proteins in a gel containing starch as substarte. c) chromatogram of Ni-NTA sepharose column (c1), chromatogram of ion exchange chromatography (c2).

*Coh 01133* and *Coh 00831* genes were PCR amplified (Fig 2A and 2B, lanes 2), cloned in pET28a, expressed in BL21 (DE3) in the presence of 1 mM IPTG at 22°C, and purified (Fig 2 and Figure A in S1 File.). Although both recombinant enzymes were capable of hydrolyzing both starch and pullulan (Table C in S1 File), they were more efficient with starch: *k*_*cat*_/*K*_*m*_ = 0.20 × 10^4^ and 16 × 10^4^ in contrast to pullulan 0.14 × 10^4^ and 0.07 × 10^4^ for *Coh 00831* enzyme and *Coh 01133* enzyme, respectively. Enzyme activities were demonstrated by zymogram analysis in the presence of pullulan (Fig 2A and 2B, lanes 7).

**Fig 3:**
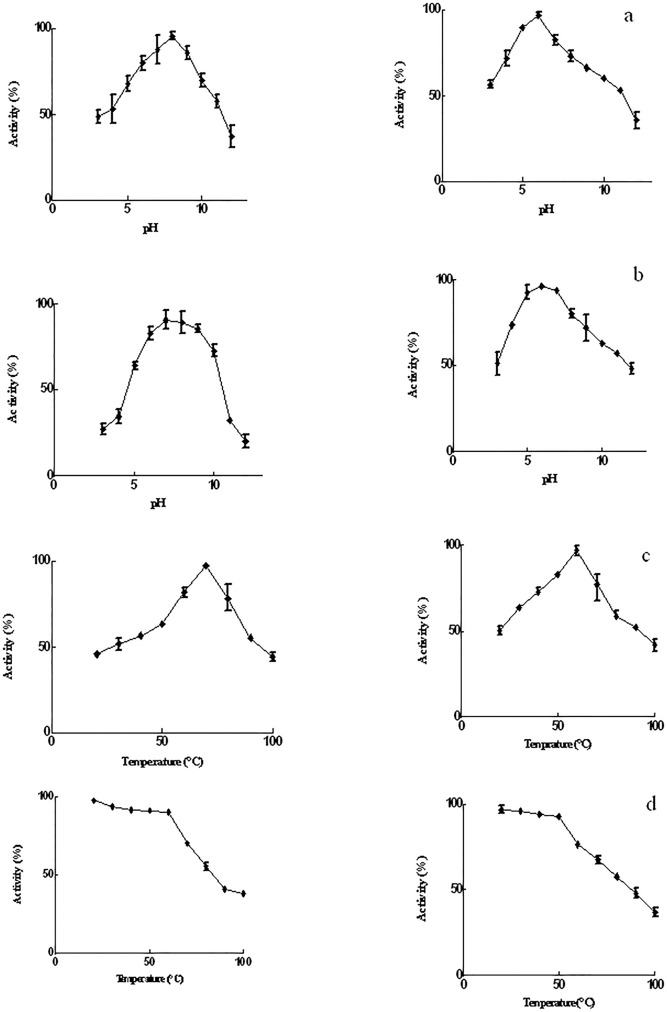
Temperature and pH characteristics of native amylopullulanases, namely *Coh01133* (left panel) and *Coh00831* (right panel), isolated from *Cohnella* sp. a) pH profile, b) pH stability, c) temperature profile, d) temperature stability.

*Coh 01133* enzyme was purified 7.09-fold, with a yield of 53% via affinity column chromatography using Ni-NTA Sepharose, and had a specific activity of 52.79 U/mg (Table 2). The enzyme had a higher relative activity at a pH range of 5–9 with optimum activity at pH = 8.0 (Fig 3A, "left panel"). The enzyme kept 92% of its activity at pHs 7.0, 8.0 and 9.0 for a duration of 90 min (Fig 3B, "left panel"). It was active at temperatures higher than 45°C up to 90°C with the optimum temperature being 70°C (Fig 3C, "left panel"). The analysis of *Coh 01133* enzyme temperature stability demonstrated that the enzyme keeps 90% of its activity at temperatures between 20–60°C for 90 min with decline at temperatures above 60°C (Fig 3D, "left panel").

**Table 2:** Purification of heterologously expressed Coh00831 and Coh01133.

Enzyme	Steps	activity (μmol/min)	Specific activity (U/mg)	Purification fold	Yield (%)
Coh01133	Pre-purification	4.87	7.44	1	100
	After purification	2.59	52.79	7.09	53
Coh00831	Pre-purification	7.79	5.15	1	100
	After purification	1.92	118.84	23.04	24

*Coh 00831* enzyme was purified 23.04-fold, with a yield of 24% via anion exchange chromatography using DEAE-Sepharose, and had a specific activity of 118.84 U/mg (Table 2). Recombinant *Coh 00831* enzyme had greater activity at pHs ranging 4–9 with optimum activity noted at pH = 6.0 (Fig 3A, right panel). The enzyme was relatively stable at pHs 5.0, 6.0, and 7.0 (Fig 3B, right panel). The enzyme was active at temperatures ranging 40–70°C with the highest activity at 60°C (Fig 3C, right panel). The enzyme is stable more than 92% in 50°C for 90 min and kept 85% of its activity at 60°C, once incubated in a range of temperatures (20–100°C) for 90 min (Fig 3D, right panel).

The enzymes’ kinetic parameters in the presence of two substrates, pullulan and starch, were calculated with Prism5. In *Coh 01133* enzyme, the K_m_ for pullulan was determined to be 0.28 mg/ml, and that for starch was 0.279 mg/ml. From the Lineweaver-Burk plot, the V_max_ values for pullulanase were calculated to be 5.099 μmol/min for pullulan and 10.45 μmol/min for starch. In *Coh 00831* enzyme, the K_m_ for pullulan was 0.21 mg/ml and for starch was 0.1898 mg/ml, and the V_max_ values were calculated to be 4.375 μmol/min for pullulan and 5.647 μmol/min for starch. The kinetic parameters (*K*_*m*_, V_max_, *k*_*cat*_ and *k*_*cat*_/*K*_*m*_) of the enzymes have been summarized in Table C in S1 File. The k_cat_ value of the *Coh 01133* enzyme toward pullulan substrate was smaller compared with *Coh 00831* enzyme. In contrast, *Coh 00831* enzyme had a lower k_cat_ value for soluble starch compared with those of *Coh 01133* enzyme (Table C in S1 File).

Afterwards, hydrolyzed products for soluble starch and pullulan with *Coh01133* and *Coh00831* recombinant enzymes were examined by HPLC analysis. The two purified enzymes hydrolyzed soluble starch to mainly maltose (G2), maltotriose (G3) and maltotetraose (G4) and hydrolyzed pullulan to maltotriose (G3) demonstrating that the enzymes show typical pullulanase activity. Neither enzyme produced glucose (G1) (Table 3).

**Table 3:** Reaction products of *Cohnella* amylopullulanase on pullulan and soluble starch. Solutions of 1% pullulan, and soluble starch were incubated at optimum temperature and pH with Coh01133 and Coh00831 enzymes. Reaction products were analyzed by HPLC for sugars.

Enzyme	Substrate	End products formed				
		Glucose	Maltose	Maltotriose	Maltotetraose	Maltohexose
Coh01133	Pullulan	0	0	100	0	0
	Soluble starch	0	29	38	30	3
Coh00831	Pullulan	0	0	100	0	0
	Soluble starch	0	25	35	29	11

### Effect of some metal ions and chemical materials

Among the metal ions and detergents tested, Mn^2+^, Ba^2+^, Ca^2+^, and Mg^2+^ improved the activity of both enzymes, while Fe^2+^, Li^2+^, Na^2+^, Triton X100, and urea caused sharp reductions in enzyme activity. More specifically, Co^2+^, Cu^2+^, and K^+^ in *Coh 01133* amylopullulanase reduced enzyme activity greatly. EDTA reduced the activities of both enzymes, specifically at 5 mM. In contrast, iodoacetamide had no major effect on enzyme activity (Table 4).

**Table 4:** The effects of cations (5 mM) and some chemical materials (a reducing agent, a chelator and detergents) on the relative activity of recombinant amylopullulanases.

Cations	Relative enzyme activity (%)	
	Coh01133	Coh00831
Control	100	100
MgCl_2_	87	193
CaCl_2_	166	169
KCl	39	76
NiCl_2_	85	84
LiCl_2_	21	30
FeCl_2_	38	55
MnCl_2_	146	171
ZnCl_2_	23	78
SrCl_2_	90	57
BaCl_2_	150	113
CuCl_2_	20	85
CoCl_2_	38	74
NaCl	32	41
Tween 20 (0.1%)	73	76
Urea (6 M)	4	2
Iodoacetamide (10 mM)	96	100
SDS (1 mM)	82	73
EDTA (2 mM)	88	96
EDTA (5 mM)	62	69
Triton X100 (0.1%)	28	35

## Discussion

Industrial enzymes have shown great promise for many aspects of human life for the last half century or so; they can improve catalytic reaction rates, considering environmental issues as opposed to chemical biocatalysts. Starch byproducts resulting from enzymatic hydrolysis have many applications in the food and pharmaceutical industries. Moreover, the use of starch hydrolyzing enzymes such as α-amylases and amylopullulanases in detergent industries compared with other enzymes has improved the efficiency of commercial enzyme complexes. It is apparent that the industry is booming in its different fields, and accordingly looking into other more efficient enzymes with greater stability. Such enzymes are generally obtained from extermophilic microorganisms like archaea, bacteria, and fungi. Among bacteria, some species of *Geobacillus* and *Bacillus* are known to produce thermophilic amylopullulanase [20–22]. Research on the thermophilic amylopullulanases is attractive not only for understanding the enzyme stability mechanisms, but also for finding better enzymes with more efficient usage in industrial process [23]. Here and in the quest for such enzymes, a native thermophilic bacterium has been isolated, namely *Cohnella* sp. A01 (accession number JN208862.1), from shrimp ponds in southern parts of Iran. Following genome sequencing (data not yet published), two amylopullulanase genes, *Coh 00831* gene and *Coh 01133* gene, were PCR amplified and used for heterologous protein expression and further biochemical characterizations. The enzymes demonstrated themselves to be thermophilic as expected from the niche from which the bacterium was isolated. The *K*_*m*_ values for the two recombinant enzymes were the lowest for pullulan and starch when compared with previously reported homologues from other bacteria (Table C in S1 File), indicating a greater affinity toward the substrates. Here, the findings are discussed for each gene separately, and the results are compared with other bacterial homologs.

*Coh 00831* amylopullulanase: Phylogenetic analysis of this protein with bacterial members of families GH13 and GH57 demonstrated that *Coh 00831* enzyme was well-grouped with GH57 and was closer to the amylopullulanases of this family (Fig 1). Almost all mesophilic amylopullulanases belong to GH13 family and the thermostable counterparts come under GH57 or GH13 family [24]. *Coh 00831* enzyme with 666 aa is among the common bacterial amylopullulanases, considering its relative molecular weight (70 kDa). The recombinant enzyme had an optimum temperature of 60°C, higher than a few previously reported bacterial amylopullulanases (Table C in S1 File). The enzyme is stable more than 92% in 50°C for 90 min. Most of thermostable amylopullulanase were active at acidic or neutral pH while *Coh 00831* amylopullulanase was active at pHs ranging 4–9, reaching an optimum at pH = 6.0. K_m_ values were illustrative of no differences between enzyme affinity toward either starch or pullulan (Table C in S1 File). However, *k*_*cat*_ was smaller for pullulan, indicating the enzyme’s preference toward starch as substrate. The purified *Coh 00831* enzyme hydrolyzed pullulan to generate G3 and hydrolyzed soluble starch to produced G2, G3, G4 and G6, which are nearly the same as those of other amylopullulanases [25–27]. Among the cations tested, Mg^2+^, Ca^2+^, and Mn^2+^ improved enzyme activity by more than 50%. The cationic inhibitors were Na^+^, K^+^, Ni^2+^, Fe^2+^, Zn^2+^, Cu^2+^, Co^2+^, and Sr^2+^ (Table 4). In general, the used detergents reduced enzyme activity. Enzyme inactivates by 6 M urea. EDTA had very little effect on the enzyme activity. Some common chelating agents used in industrial cleaning compounds include EDTA, phosphates and sodium citrate. Iodoacetamide did not inactivate the enzyme, indicating no cysteine residue within the enzyme active site (Table 4).

*Coh 01133* amylopullulanase: Phylogenetic analysis of this protein, similar to *Coh 00831* enzyme, grouped the protein sequence with GH57, next to other amylopullulanases (Fig 1) similar to mentioned above. *Coh 01133* enzyme with 1226 aa and a relative mass of 125 kDa is among the large amylopullulanases of GH57, similar to proteins from *Thermococus* Sp. (Table C in S1 File). The enzyme’s optimum temperature was 70°C; accordingly, it can be classified as a thermophilic amylopullulanase. However, compared to other previously characterized amylopullulanases, this enzyme falls within the mid-range. It is suggested that thorough comparative studies with other known GH57 members (Table C in S1 File) be conducted toward engineering this enzyme to improve the optimal temperature. Given that most amylolitic industrial enzymes need to be active at temperatures higher than 50°C, *Coh 01133* amylopullulanase can be considered a suitable industrial enzyme. The enzyme is stable more than 90% in 60°C for 90 min. Earlier studies were indicative of a pH stability of 5.5–6 for most pullulanase in GH57 [28–31]. In this study, the enzyme demonstrated an optimum pH of 8.0 with wide pH stability (5.0–9.0) that makes it suitable for use in the detergent industry. K_m_ values for *Coh 01133* enzyme (~ 0.28) shown in Table C in S1 File. However, *k*_*cat*_ and *k*_*cat*_/*K*_*m*_ values were greater for starch than pullulan, indicating that starch is a better substrate for the enzyme (Table C in S1 File). The action of the enzyme on pullulan and soluble starch formed G3 and G2, G3, and G4 respectively. Similar results have been observed for the other amylopullulanases [25–27]. Cations such as Mn^2+^, Ba^2+^ Ca^2+^, and Mg^2+^ improved the *Coh 01133* amylopullulanase activity; for Ca^2+^ and Mg^2+^ up to 2-fold activity was noted. The improvement of amylopullulanase activity in the presence of Ca^2+^ has been reported elsewhere; for instance, *Micrococcus* sp. pullulan hydrolyzing enzyme was activated after CaCl_2_ addition [28, 32–34]. Additionally, Ca^2+^ and Mg^2+^ improved the temperature stability of the enzyme [35, 36]. Some other cations, Li^+^, Na^+^, K^+^, Co^2+^, and Cu^2+^, severely reduced enzyme activity. Similar reductions were reported earlier, for example amylopullulanases activity of *T*. *hydrothermalis* did not affect by Na^+^ and Mg^2+^ while Mn^2+^ increase its activity. Metal cations inhibitory effect such as Ni^2+^ and Cu^2+^ has been seen for almost all amylopullulanases [6, 18, 28, 31, 36, 37].

## Conclusion

Two amylopullulanse genes from an endogenous bacterium belonging to GH57, namely *Coh 00831* (~70 kDa) and *Coh 01133*, (~127 kDa), were PCR amplified and heterologously expressed in *E*. *coli*. Recombinant enzymes were assayed against pullulan and starch and demonstrated greater affinity toward starch. Thus, both enzymes have the potential to be used for liquefaction in the starch industry. The effects of metal divalent cations were studied, and Ca^2+^, Mg^2+^, Mn^2+^, and Ba^2+^ were demonstrated to improve enzyme activity by more than 50%.

## Supporting information

S1 File Table A in S1 File: Bacterial α-amylases and amylopullulanases used for multiple sequence alignment and phylogenetic tree. Table B in S1 File: The % identity of proteins from this study with other Amylopullulanase. Table C in S1 File: Molecular weight, pH optimum, temperature optimum and Kinetic parameters of some amylopullulanases from GH13 and GH57. Figure A in S1 File: Chromatogram of Ni-NTA sepharose column (c1), chromatogram of ion exchange chromatography (c2)
(PPTX)
