# Supplementary material for: Cohnella amylopullulanases: Biochemical characterization of two recombinant thermophilic enzymes
Source: PLoS One. 2017 Apr 10;12(4):e0175013. doi: 10.1371/journal.pone.0175013 (PMC5386253; doi:10.1371/journal.pone.0175013)
Supplement: S1 File — Table A in S1 File: Bacterial α-amylases and amylopullulanases used for multiple sequence alignment and phylogenetic tree. Table B in S1 File: The % identity of proteins from this study with other Amylopullulanase. Table C in S1 File: Molecular weight, pH optimum, temperature optimum and Kinetic parameters of some amylopullulanases from GH13 and GH57. Figure A in S1 File: Chromatogram of Ni-NTA sepharose column (c1), chromatogram of ion exchange chromatography (c2) (PPTX) [file pone.0175013.s001.pptx]

## Slide 1
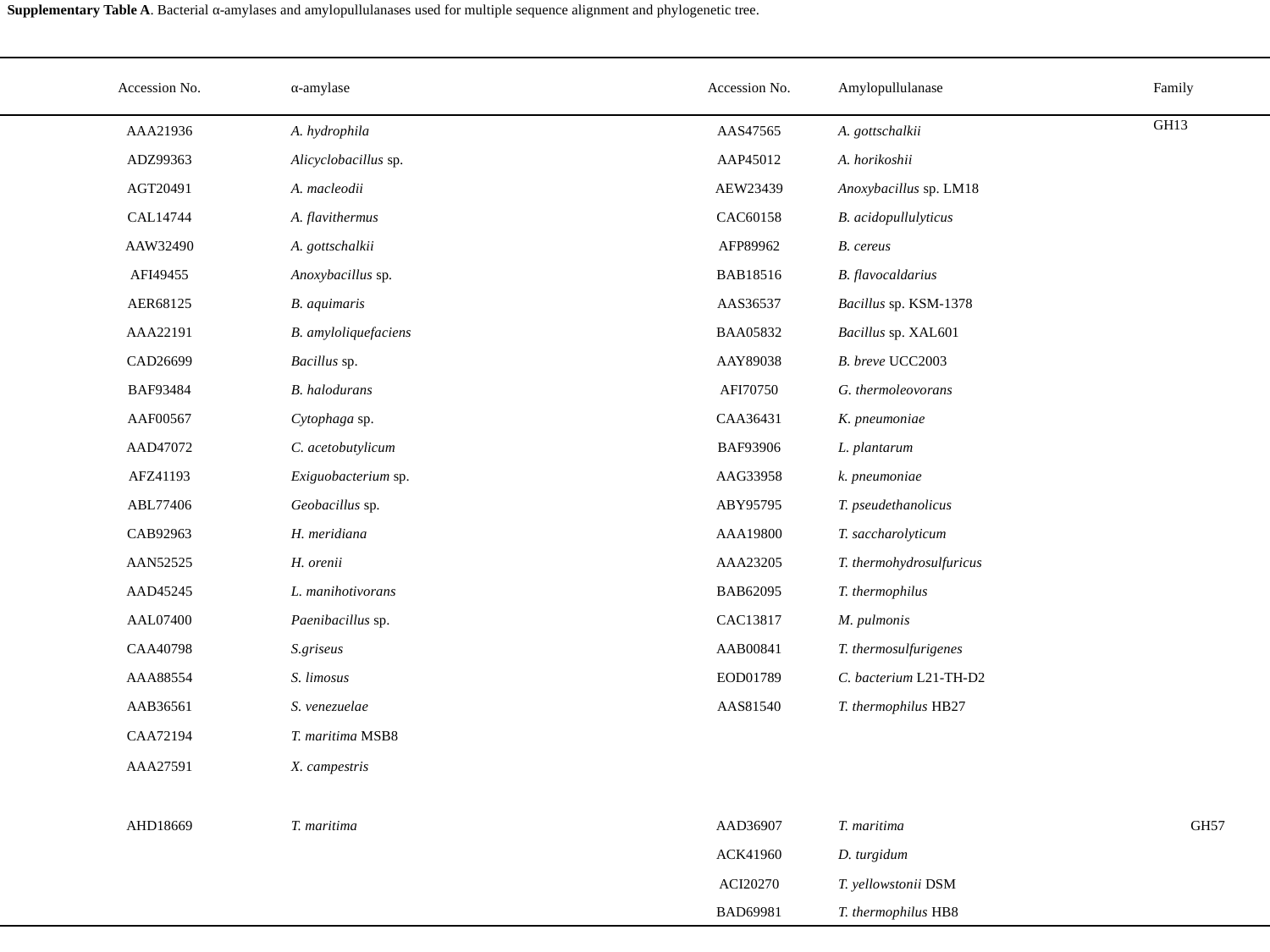

| Supplementary Table A. Bacterial α-amylases and amylopullulanases used for multiple sequence alignment and phylogenetic tree. | | | | | | |
| --- | --- | --- | --- | --- | --- | --- |
| | Accession No. | α-amylase | | Accession No. | Amylopullulanase | Family |
| | AAA21936 | A. hydrophila | | AAS47565 | A. gottschalkii | GH13 |
| | ADZ99363 | Alicyclobacillus sp. | | AAP45012 | A. horikoshii | |
| | AGT20491 | A. macleodii | | AEW23439 | Anoxybacillus sp. LM18 | |
| | CAL14744 | A. flavithermus | | CAC60158 | B. acidopullulyticus | |
| | AAW32490 | A. gottschalkii | | AFP89962 | B. cereus | |
| | AFI49455 | Anoxybacillus sp. | | BAB18516 | B. flavocaldarius | |
| | AER68125 | B. aquimaris | | AAS36537 | Bacillus sp. KSM-1378 | |
| | AAA22191 | B. amyloliquefaciens | | BAA05832 | Bacillus sp. XAL601 | |
| | CAD26699 | Bacillus sp. | | AAY89038 | B. breve UCC2003 | |
| | BAF93484 | B. halodurans | | AFI70750 | G. thermoleovorans | |
| | AAF00567 | Cytophaga sp. | | CAA36431 | K. pneumoniae | |
| | AAD47072 | C. acetobutylicum | | BAF93906 | L. plantarum | |
| | AFZ41193 | Exiguobacterium sp. | | AAG33958 | k. pneumoniae | |
| | ABL77406 | Geobacillus sp. | | ABY95795 | T. pseudethanolicus | |
| | CAB92963 | H. meridiana | | AAA19800 | T. saccharolyticum | |
| | AAN52525 | H. orenii | | AAA23205 | T. thermohydrosulfuricus | |
| | AAD45245 | L. manihotivorans | | BAB62095 | T. thermophilus | |
| | AAL07400 | Paenibacillus sp. | | CAC13817 | M. pulmonis | |
| | CAA40798 | S.griseus | | AAB00841 | T. thermosulfurigenes | |
| | AAA88554 | S. limosus | | EOD01789 | C. bacterium L21-TH-D2 | |
| | AAB36561 | S. venezuelae | | AAS81540 | T. thermophilus HB27 | |
| | CAA72194 | T. maritima MSB8 | | | | |
| | AAA27591 | X. campestris | | | | |
| | | | | | | |
| | AHD18669 | T. maritima | | AAD36907 | T. maritima | GH57 |
| | | | | ACK41960 | D. turgidum | |
| | | | | ACI20270 | T. yellowstonii DSM | |
| | | | | BAD69981 | T. thermophilus HB8 | |

## Slide 2
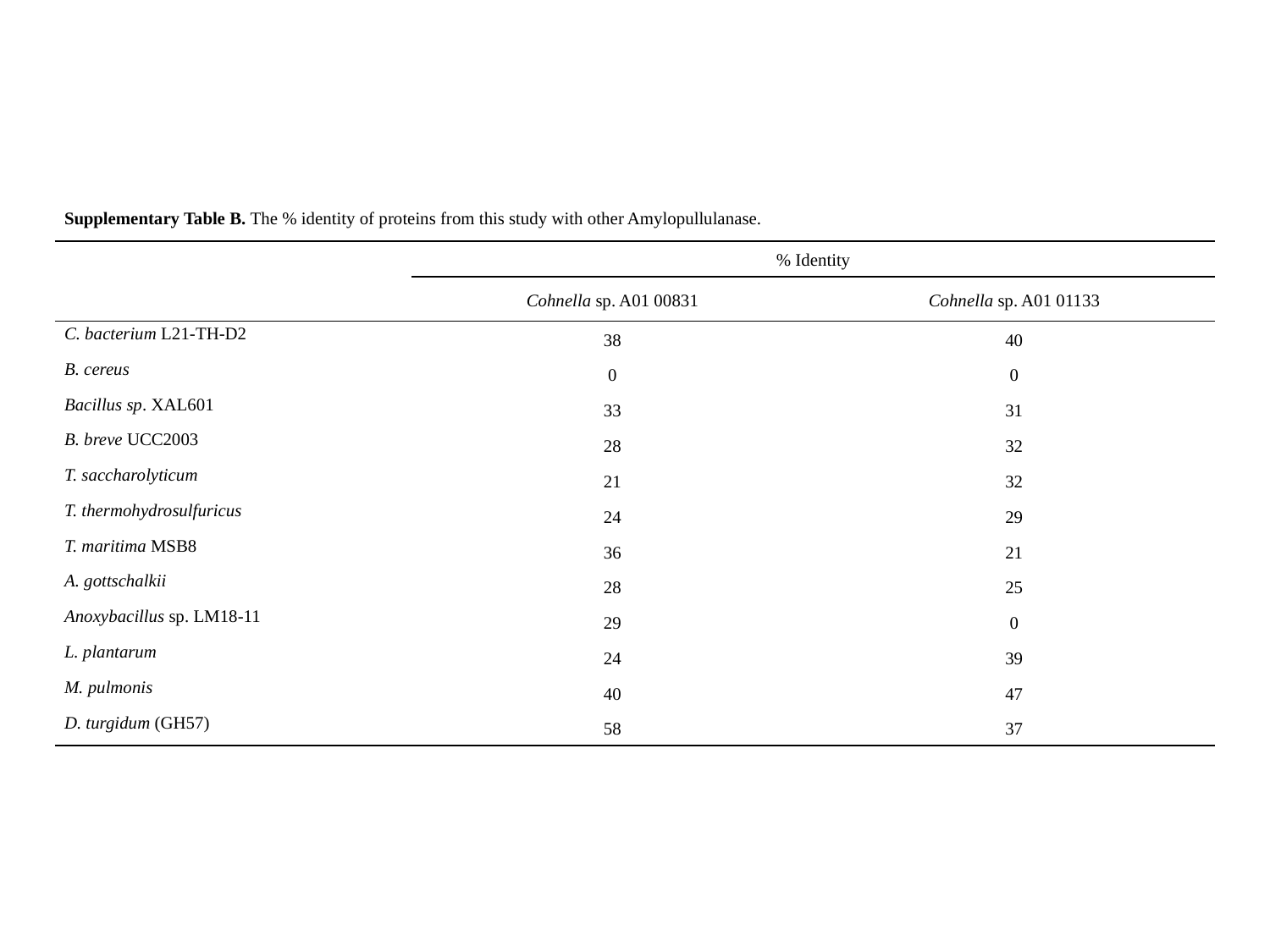

| Supplementary Table B. The % identity of proteins from this study with other Amylopullulanase. | | |
| --- | --- | --- |
| | % Identity | |
| | Cohnella sp. A01 00831 | Cohnella sp. A01 01133 |
| C. bacterium L21-TH-D2 | 38 | 40 |
| B. cereus | 0 | 0 |
| Bacillus sp. XAL601 | 33 | 31 |
| B. breve UCC2003 | 28 | 32 |
| T. saccharolyticum | 21 | 32 |
| T. thermohydrosulfuricus | 24 | 29 |
| T. maritima MSB8 | 36 | 21 |
| A. gottschalkii | 28 | 25 |
| Anoxybacillus sp. LM18-11 | 29 | 0 |
| L. plantarum | 24 | 39 |
| M. pulmonis | 40 | 47 |
| D. turgidum (GH57) | 58 | 37 |

## Slide 3
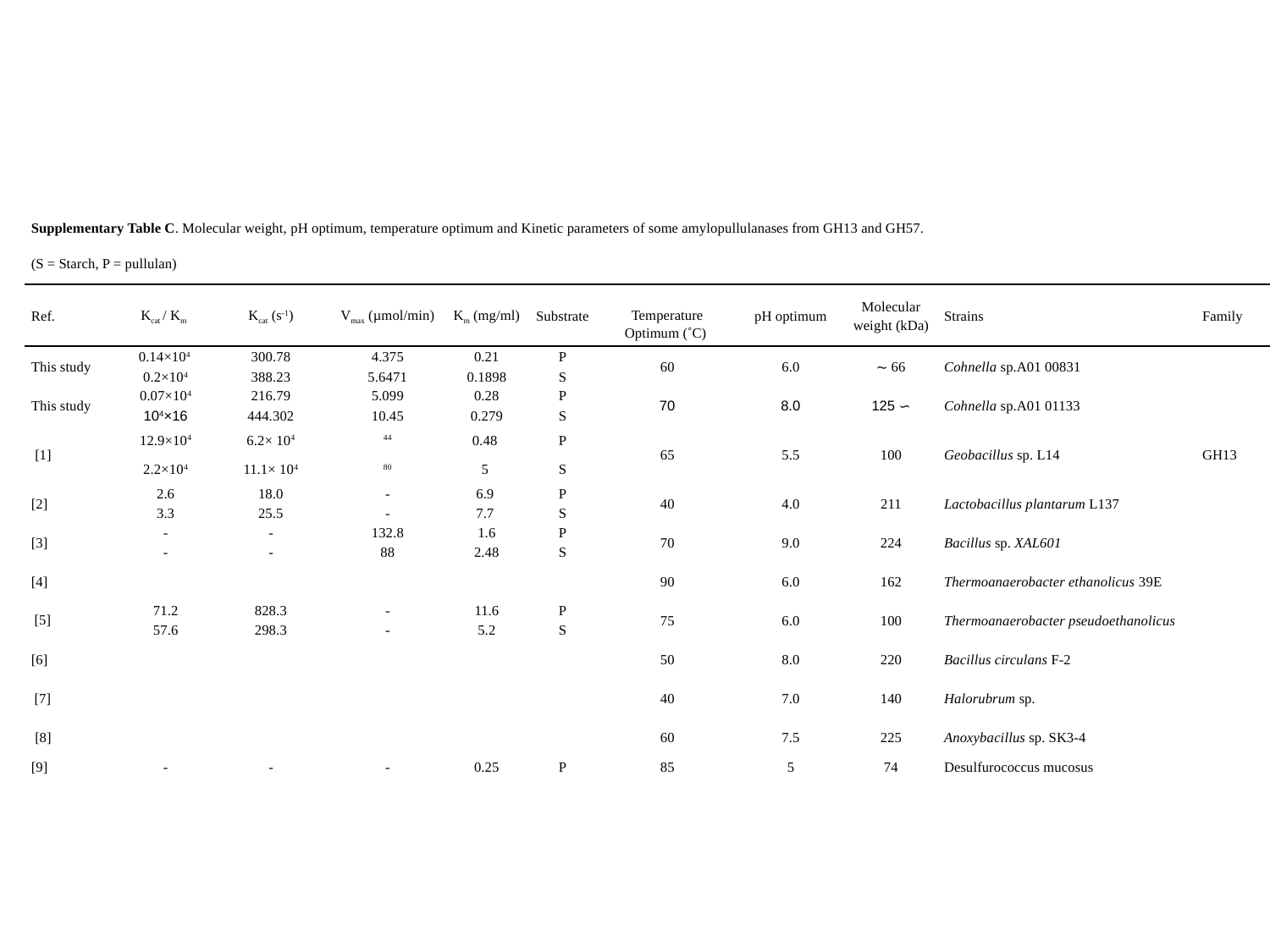

| Supplementary Table C. Molecular weight, pH optimum, temperature optimum and Kinetic parameters of some amylopullulanases from GH13 and GH57. (S = Starch, P = pullulan) | | | | | | | | | | |
| --- | --- | --- | --- | --- | --- | --- | --- | --- | --- | --- |
| Ref. | Kcat / Km | Kcat (s-1) | Vmax (µmol/min) | Km (mg/ml) | Substrate | Temperature Optimum (˚C) | pH optimum | Molecular weight (kDa) | Strains | Family |
| This study | 0.14×10^4^ | 300.78 | 4.375 | 0.21 | P | 60 | 6.0 | ∼ 66 | Cohnella sp.A01 00831 | |
| | 0.2×10^4^ | 388.23 | 5.6471 | 0.1898 | S | | | | | |
| This study | 0.07×10^4^ | 216.79 | 5.099 | 0.28 | P | 70 | 8.0 | ∼ 125 | Cohnella sp.A01 01133 | |
| | 16×104 | 444.302 | 10.45 | 0.279 | S | | | | | |
| [1] | 12.9×104 | 6.2× 10^4^ | 44 | 0.48 | P | 65 | 5.5 | 100 | Geobacillus sp. L14 | GH13 |
| | 2.2×10^4^ | 11.1× 104 | 80 | 5 | S | | | | | |
| [2] | 2.6 | 18.0 | - | 6.9 | P | 40 | 4.0 | 211 | Lactobacillus plantarum L137 | |
| | 3.3 | 25.5 | - | 7.7 | S | | | | | |
| [3] | - | - | 132.8 | 1.6 | P | 70 | 9.0 | 224 | Bacillus sp. XAL601 | |
| | - | - | 88 | 2.48 | S | | | | | |
| [4] | | | | | | 90 | 6.0 | 162 | Thermoanaerobacter ethanolicus 39E | |
| | | | | | | | | | | |
| [5] | 71.2 | 828.3 | - | 11.6 | P | 75 | 6.0 | 100 | Thermoanaerobacter pseudoethanolicus | |
| | 57.6 | 298.3 | - | 5.2 | S | | | | | |
| [6] | | | | | | 50 | 8.0 | 220 | Bacillus circulans F-2 | |
| | | | | | | | | | | |
| [7] | | | | | | 40 | 7.0 | 140 | Halorubrum sp. | |
| | | | | | | | | | | |
| [8] | | | | | | 60 | 7.5 | 225 | Anoxybacillus sp. SK3-4 | |
| | | | | | | | | | | |
| [9] | - | - | - | 0.25 | P | 85 | 5 | 74 | Desulfurococcus mucosus | |

## Slide 4
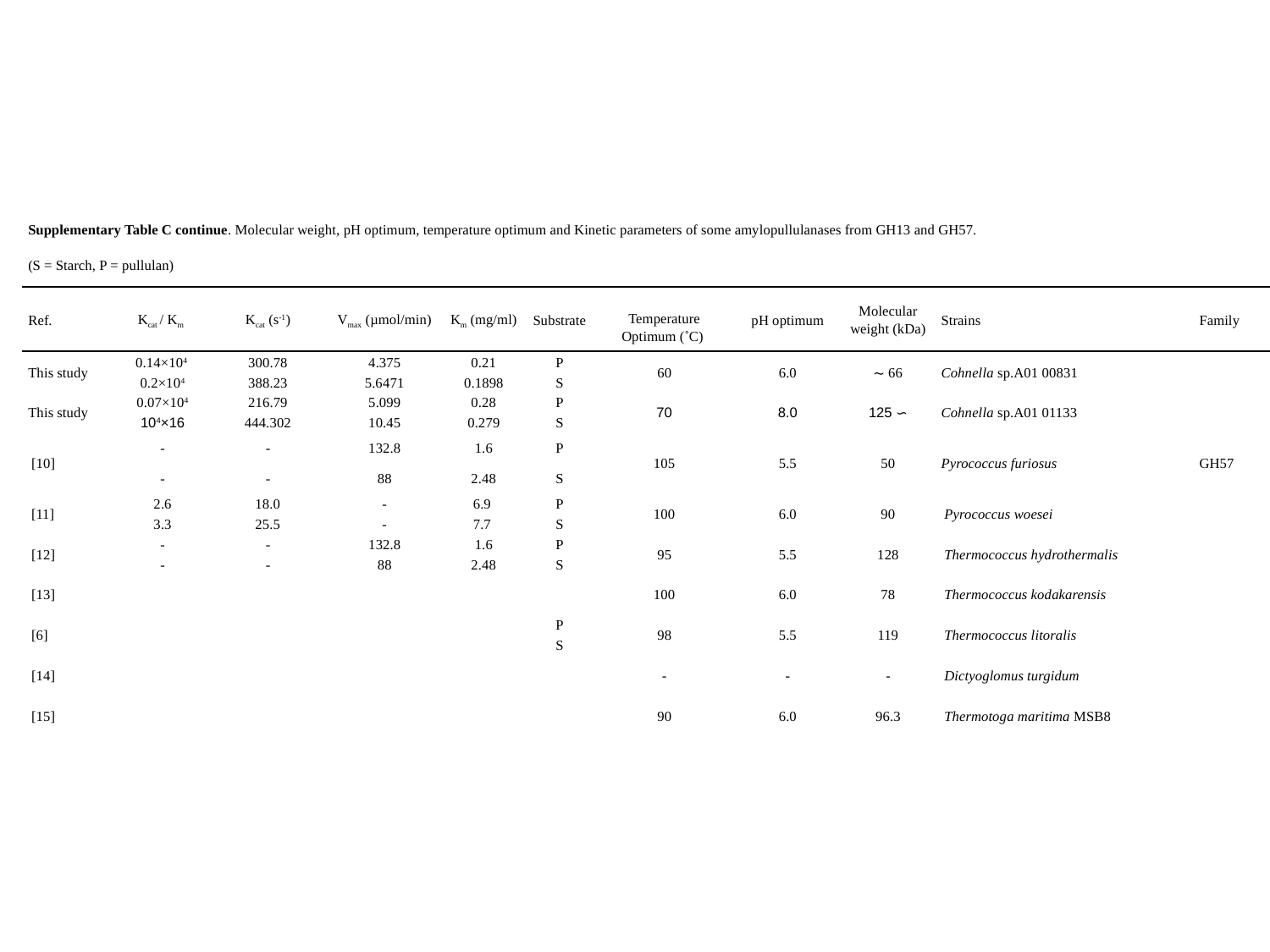

| Supplementary Table C continue. Molecular weight, pH optimum, temperature optimum and Kinetic parameters of some amylopullulanases from GH13 and GH57. (S = Starch, P = pullulan) | | | | | | | | | | |
| --- | --- | --- | --- | --- | --- | --- | --- | --- | --- | --- |
| Ref. | Kcat / Km | Kcat (s-1) | Vmax (µmol/min) | Km (mg/ml) | Substrate | Temperature Optimum (˚C) | pH optimum | Molecular weight (kDa) | Strains | Family |
| This study | 0.14×10^4^ | 300.78 | 4.375 | 0.21 | P | 60 | 6.0 | ∼ 66 | Cohnella sp.A01 00831 | |
| | 0.2×10^4^ | 388.23 | 5.6471 | 0.1898 | S | | | | | |
| This study | 0.07×10^4^ | 216.79 | 5.099 | 0.28 | P | 70 | 8.0 | ∼ 125 | Cohnella sp.A01 01133 | |
| | 16×104 | 444.302 | 10.45 | 0.279 | S | | | | | |
| [10] | - | - | 132.8 | 1.6 | P | 105 | 5.5 | 50 | Pyrococcus furiosus | GH57 |
| | - | - | 88 | 2.48 | S | | | | | |
| [11] | 2.6 | 18.0 | - | 6.9 | P | 100 | 6.0 | 90 | Pyrococcus woesei | |
| | 3.3 | 25.5 | - | 7.7 | S | | | | | |
| [12] | - | - | 132.8 | 1.6 | P | 95 | 5.5 | 128 | Thermococcus hydrothermalis | |
| | - | - | 88 | 2.48 | S | | | | | |
| [13] | | | | | | 100 | 6.0 | 78 | Thermococcus kodakarensis | |
| | | | | | | | | | | |
| [6] | | | | | P | 98 | 5.5 | 119 | Thermococcus litoralis | |
| | | | | | S | | | | | |
| [14] | | | | | | - | - | - | Dictyoglomus turgidum | |
| | | | | | | | | | | |
| [15] | | | | | | 90 | 6.0 | 96.3 | Thermotoga maritima MSB8 | |
| | | | | | | | | | | |
| | | | | | | | | | | |
| | | | | | | | | | | |
| | | | | | | | | | | |

## Slide 5
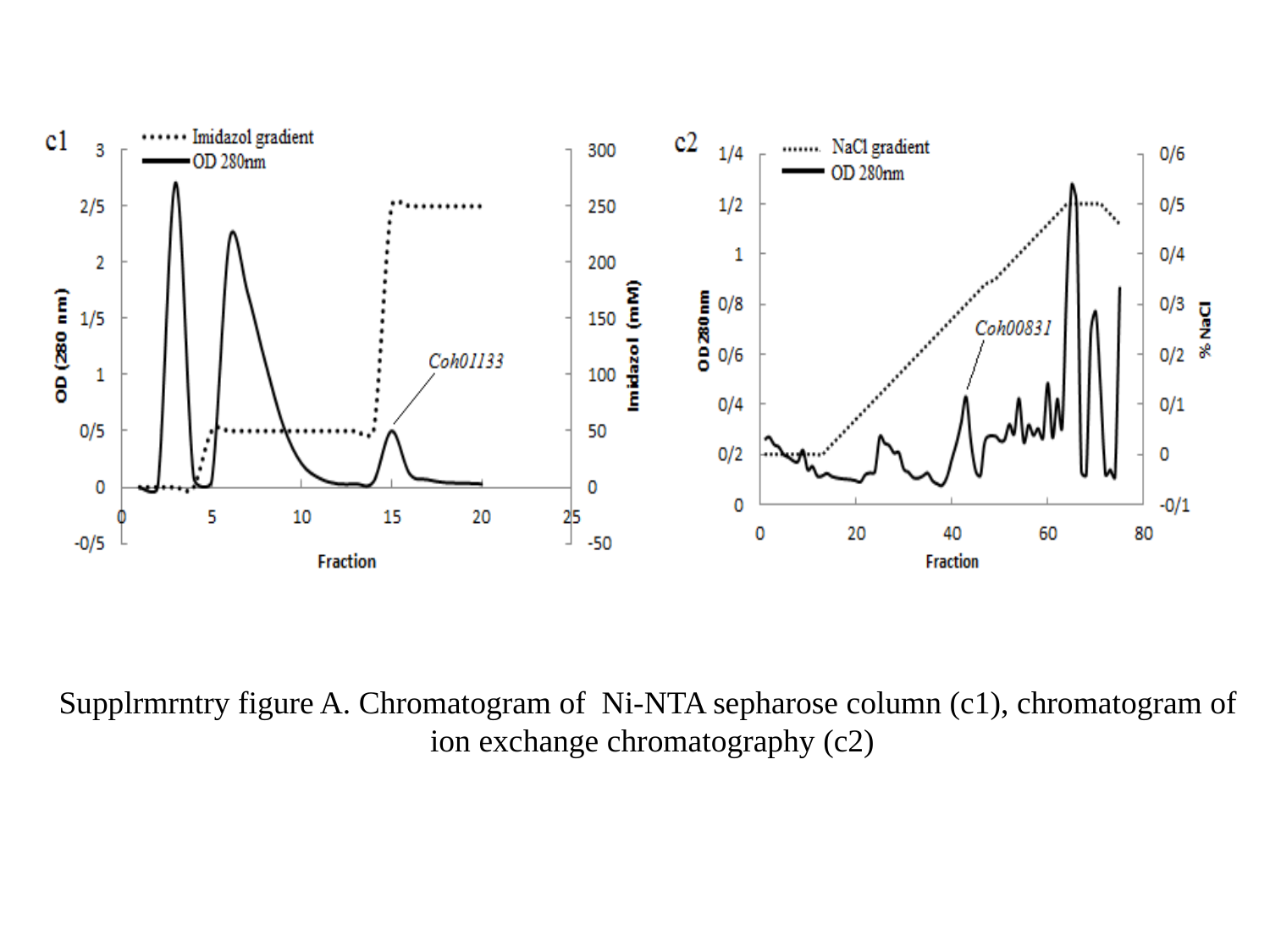

Supplrmrntry figure A. Chromatogram of Ni-NTA sepharose column (c1), chromatogram of ion exchange chromatography (c2)

## Slide 6
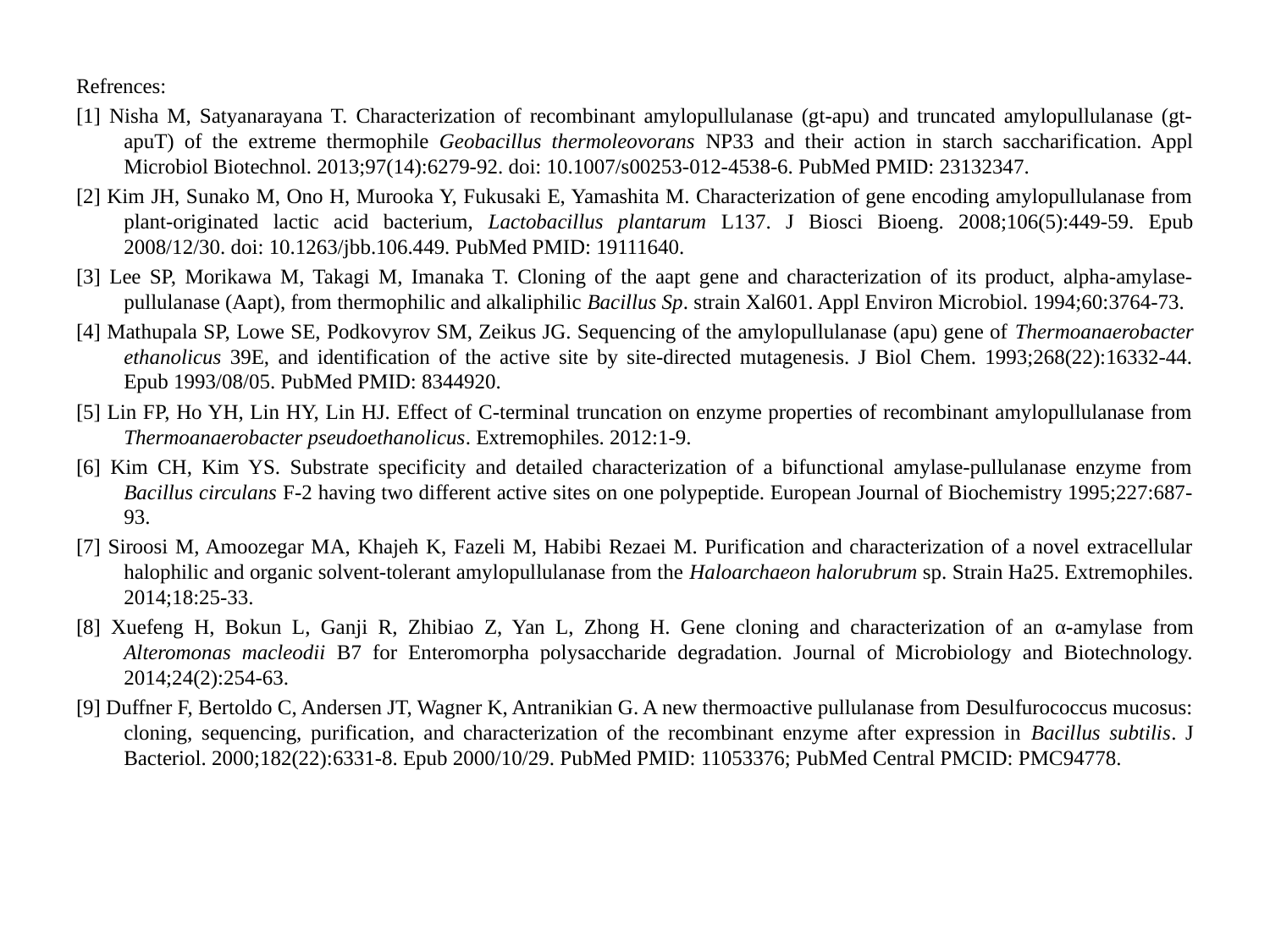

Refrences:
[1] Nisha M, Satyanarayana T. Characterization of recombinant amylopullulanase (gt-apu) and truncated amylopullulanase (gt-apuT) of the extreme thermophile Geobacillus thermoleovorans NP33 and their action in starch saccharification. Appl Microbiol Biotechnol. 2013;97(14):6279-92. doi: 10.1007/s00253-012-4538-6. PubMed PMID: 23132347.
[2] Kim JH, Sunako M, Ono H, Murooka Y, Fukusaki E, Yamashita M. Characterization of gene encoding amylopullulanase from plant-originated lactic acid bacterium, Lactobacillus plantarum L137. J Biosci Bioeng. 2008;106(5):449-59. Epub 2008/12/30. doi: 10.1263/jbb.106.449. PubMed PMID: 19111640.
[3] Lee SP, Morikawa M, Takagi M, Imanaka T. Cloning of the aapt gene and characterization of its product, alpha-amylase-pullulanase (Aapt), from thermophilic and alkaliphilic Bacillus Sp. strain Xal601. Appl Environ Microbiol. 1994;60:3764-73.
[4] Mathupala SP, Lowe SE, Podkovyrov SM, Zeikus JG. Sequencing of the amylopullulanase (apu) gene of Thermoanaerobacter ethanolicus 39E, and identification of the active site by site-directed mutagenesis. J Biol Chem. 1993;268(22):16332-44. Epub 1993/08/05. PubMed PMID: 8344920.
[5] Lin FP, Ho YH, Lin HY, Lin HJ. Effect of C-terminal truncation on enzyme properties of recombinant amylopullulanase from Thermoanaerobacter pseudoethanolicus. Extremophiles. 2012:1-9.
[6] Kim CH, Kim YS. Substrate specificity and detailed characterization of a bifunctional amylase‐pullulanase enzyme from Bacillus circulans F‐2 having two different active sites on one polypeptide. European Journal of Biochemistry 1995;227:687-93.
[7] Siroosi M, Amoozegar MA, Khajeh K, Fazeli M, Habibi Rezaei M. Purification and characterization of a novel extracellular halophilic and organic solvent-tolerant amylopullulanase from the Haloarchaeon halorubrum sp. Strain Ha25. Extremophiles. 2014;18:25-33.
[8] Xuefeng H, Bokun L, Ganji R, Zhibiao Z, Yan L, Zhong H. Gene cloning and characterization of an α-amylase from Alteromonas macleodii B7 for Enteromorpha polysaccharide degradation. Journal of Microbiology and Biotechnology. 2014;24(2):254-63.
[9] Duffner F, Bertoldo C, Andersen JT, Wagner K, Antranikian G. A new thermoactive pullulanase from Desulfurococcus mucosus: cloning, sequencing, purification, and characterization of the recombinant enzyme after expression in Bacillus subtilis. J Bacteriol. 2000;182(22):6331-8. Epub 2000/10/29. PubMed PMID: 11053376; PubMed Central PMCID: PMC94778.

## Slide 7
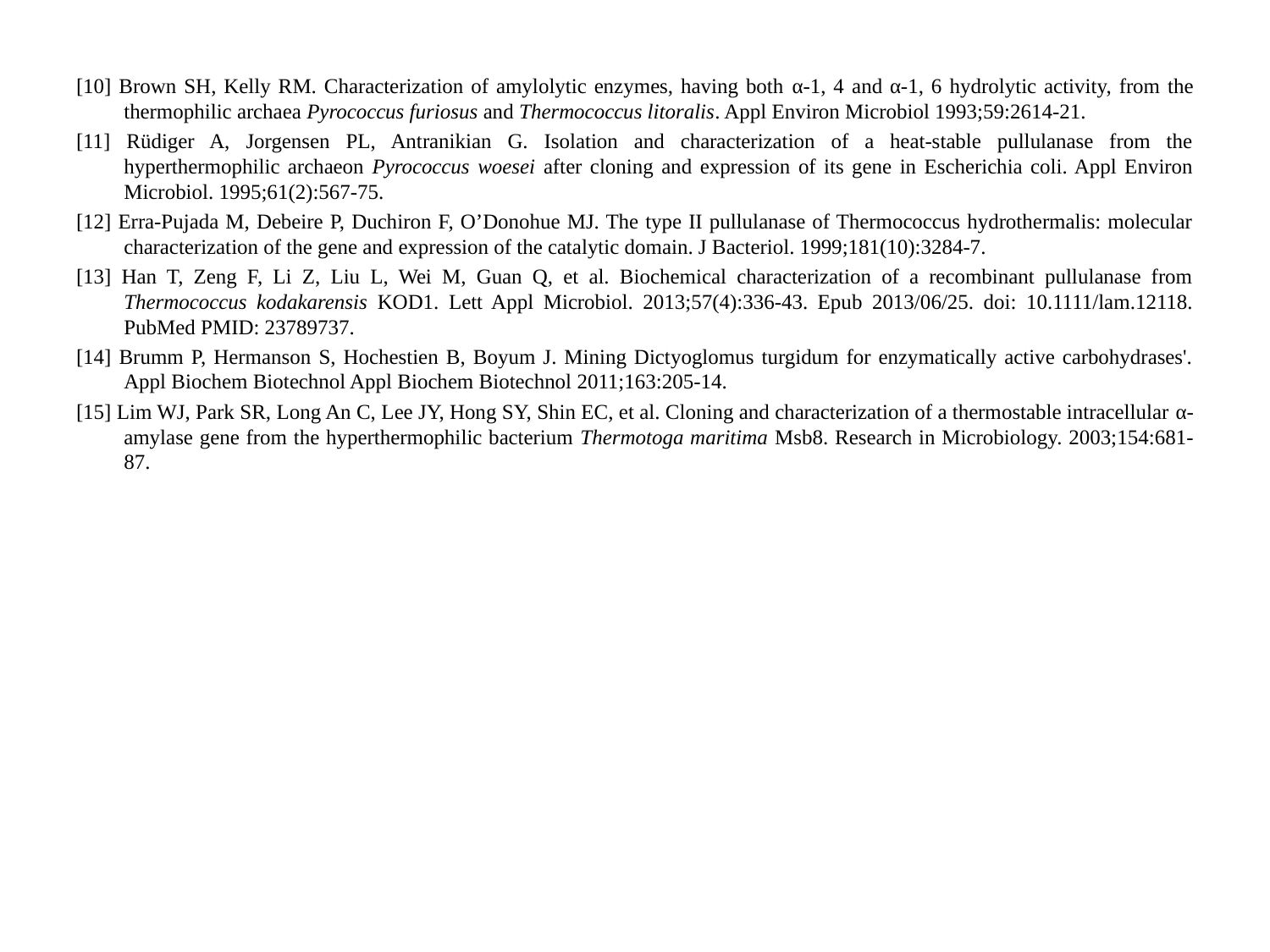

[10] Brown SH, Kelly RM. Characterization of amylolytic enzymes, having both α-1, 4 and α-1, 6 hydrolytic activity, from the thermophilic archaea Pyrococcus furiosus and Thermococcus litoralis. Appl Environ Microbiol 1993;59:2614-21.
[11] Rüdiger A, Jorgensen PL, Antranikian G. Isolation and characterization of a heat-stable pullulanase from the hyperthermophilic archaeon Pyrococcus woesei after cloning and expression of its gene in Escherichia coli. Appl Environ Microbiol. 1995;61(2):567-75.
[12] Erra-Pujada M, Debeire P, Duchiron F, O’Donohue MJ. The type II pullulanase of Thermococcus hydrothermalis: molecular characterization of the gene and expression of the catalytic domain. J Bacteriol. 1999;181(10):3284-7.
[13] Han T, Zeng F, Li Z, Liu L, Wei M, Guan Q, et al. Biochemical characterization of a recombinant pullulanase from Thermococcus kodakarensis KOD1. Lett Appl Microbiol. 2013;57(4):336-43. Epub 2013/06/25. doi: 10.1111/lam.12118. PubMed PMID: 23789737.
[14] Brumm P, Hermanson S, Hochestien B, Boyum J. Mining Dictyoglomus turgidum for enzymatically active carbohydrases'. Appl Biochem Biotechnol Appl Biochem Biotechnol 2011;163:205-14.
[15] Lim WJ, Park SR, Long An C, Lee JY, Hong SY, Shin EC, et al. Cloning and characterization of a thermostable intracellular α-amylase gene from the hyperthermophilic bacterium Thermotoga maritima Msb8. Research in Microbiology. 2003;154:681-87.
